# Psychometric properties of the Clinical Sustainability Assessment Tool (CSAT) short form across three research centers evaluating effectiveness and implementation of a cancer symptom surveillance and management intervention

**DOI:** 10.1186/s43058-026-00867-5

**Published:** 2026-01-29

**Authors:** James L. Merle, Maja Kuharic, David Cella, Sandra A. Mitchell, Jessica D. Austin, Jennifer L. Ridgeway, Michael J. Hassett, Roshan Paudel, Ann Marie Flores, Lisa DiMartino, Wynne E. Norton, Andrea L. Cheville, Justin D. Smith, the IMPACT Consortium, Andrea L. Cheville, Raymond U. Osarogiagbon, Deborah Schrag, Sandra L. Wong, Barbara L. Kroner, Ashley Wilder Smith, Sofia Garcia, Joan Griffin, Roxanne Jensen, Kathryn Ruddy, Betina Yanez, Jessica J. Bian, Don S. Dizon, Hannah W. Hazard-Jenkins, Mary-Anne Ardini, Paige Ahrens, Michael Bass, Megan Begnoche, September Cahue, Kimberly Caron, Linda Chlan, Ava Coughlin, Christine Cronin, Samira Dias, Nicolas Faris, Martha Garcia, Karla Hemming, Jeph Herrin, Christine Hodgdon, Sheetal Kircher, Kurt Kroenke, Veronica Lam, Nicola Lancki, Quan H. Mai, Jennifer Mallow, Nadine J. McCleary, Mary O’Connor, Deirdre Pachman, Loretta Pearson, Frank Penedo, Jewel Podratz, Jennifer Popovic, Liliana Preiss, Parvez Rahman, Sarah Redmond, James Reich, Joshua Richardson, Kimberly Richardson, Karen Schaepe, Tiana Poirier-Shelton, Philip Silberman, Jaclyn Simpson, Laura Tasker, Nathan Tesch, Cindy Tofthagen, Angela Tramontano, Benjamin D. Tyndall, Hajime Uno, Firas Wehbe, Bryan Weiner

**Affiliations:** 1 Intermountain Healthcare Department of Population Health Sciences, Spencer Fox Eccles School of Medicine at the University of Utah, 295 Chipeta Way, Salt Lake City, UT 84108 USA; 2 Department of Medical Social Sciences, Northwestern University Feinberg School of Medicine and Robert H. Lurie Comprehensive Cancer Center, Northwestern University, Chicago, IL USA; 3 Division of Cancer Control and Population Sciences, National Cancer Institute, Bethesda, MD USA; 4 Division of Epidemiology, Department of Quantitative Health Sciences, Mayo Clinic, Scottsdale, AZ USA; 5 Robert D. and Patricia E. Kern Center for the Science of Health Care Delivery and Division of Health Care Delivery Research, Mayo Clinic, Rochester, MN USA; 6 Departments of Medical Oncology and Quality & Patient Safety, Dana-Farber Cancer Institute, Boston, MA USA; 7 Dana-Farber Cancer Institute, Boston, MA USA; 8 Department of Physical Therapy and Human Movement Sciences, Feinberg School of Medicine, Northwestern University, Chicago, IL USA; 9 Peter O’Donnell Jr. School of Public Health, University of Texas Southwestern Medical Center, Dallas, TX USA; 10 Department of Physical Medicine and Rehabilitation, Mayo Clinic, Rochester, MN USA

**Keywords:** Factor Analysis, Clinical Sustainability Assessment Tool, Item Response Theory, Confirmatory Factor Analysis, Cancer, Implementation Science

## Abstract

**Objective:**

The Clinical Sustainability Assessment Tool (CSAT) is designed to capture determinants of sustainable clinical practices over time. Although the full 49-item CSAT instrument has demonstrated strong psychometric properties, the 21-item short form has had limited evaluation. This study aimed to assess the CSAT short form (CSAT Short) across different respondent characteristics and care delivery settings.

**Methods:**

We evaluated the CSAT Short in a sample of healthcare personnel (*N* = 256 respondents) drawn from across three hybrid effectiveness-implementation studies in a research consortium, all of which tested routine symptom surveillance and integration of symptom management interventions in ambulatory oncology care settings in the US. Confirmatory factor analyses (CFA) and mIRT were conducted to assess the CSAT Short's fit to the hypothesized factor structure. Multiple-group CFA was used to test for measurement invariance across groups of respondents with different professional roles, years in current role, and different work settings.

**Results:**

The hypothesized seven factor structure of the CSAT Short exhibited good fit to the data and strong internal consistency in our sample of healthcare personnel drawn from across three large pragmatic trials (CFI = .99,TLI = .98,*X*^2^(182) = 658.99,*p* < .001;SRMR = .031,RMSEA = .10). Tests of measurement invariance indicated the respondent’s role in the clinical setting (i.e., clinician vs. non-clinician) and years in current role (< 10 years vs. ≥10 years) were invariant. However, significant variance was found between respondents from three different Research Centers within the IMPACT consortium. The second-order mIRT model demonstrated acceptable fit based on most indices (M2(56) = 148.69, *p* < .001; RMSEA = 0.059, 90% CI[0.048, 0.071];SRMSR = 0.057; CFI = 0.917), though the TLI (0.845) was below the recommended threshold. Item-level fit varied, with RMSEA *S-X*^*2*^ values indicating six items had acceptable fit, nine items had marginal fit, and five items had poor fit.

**Conclusions:**

The CSAT Short is recommended to assess sustainability in oncology settings, though users should be cautious when comparing scores across different healthcare systems. Tests of invariance were nonsignificant except for variance by Research Center. Despite some items exhibiting suboptimal fit in mIRT, the overall model fit and reliability were strong. This study advances our understanding of sustainability measurement and the applicability of the CSAT Short across implementation settings and respondents.

**Supplementary Information:**

The online version contains supplementary material available at 10.1186/s43058-026-00867-5.

Contributions to the Literature

- This study provides additional psychometric evidence supporting the use of the CSAT Short among healthcare personnel participating in oncology-based hybrid-effectiveness implementation trials
- Findings indicate that the CSAT Short produces reliable and valid scores when used among respondents with different professional roles and varying years in their current role within similar implementation contexts
- This study highlights potential contextual variation in measurement performance across healthcare systems, underscoring the importance of caution when comparing CSAT Short scores across organizational settings

## Background

Sustainability is defined as the continued delivery of an evidence-based intervention or program at a sufficient level to maintain its intended benefits over time [1]. Despite the growing recognition of its importance among researchers and practitioners, sustainment of evidence-based interventions (EBIs) remains a significant challenge in many healthcare settings and is often not achieved in practice [2, 3]. The long-term sustainability of EBIs is essential for achieving lasting improvements in health outcomes, but it is influenced by various factors, including organizational capacity, leadership support, stakeholder engagement, and the fit between the intervention and the local context [4]. Sustainability and related constructs (e.g., sustainment, maintenance, institutionalization) are conceptualized in various ways, such as maintaining program activities, demonstrating enduring favorable effects on population health outcomes, institutionalizing a program within an organization, and building the capacity for continued adaptation and evolution [5, 6]. The diversity in how the field conceptualizes sustainability has led to the development of several sustainability frameworks and measures, each with their own strengths and limitations [7]. Some frameworks focus on individual-level factors that influence the adoption and maintenance of EBIs, while others emphasize organizational and system-level determinants of sustainability [8]. To advance research on sustainability of EBIs, rigorous and systematic psychometric evaluation of measures is needed across care delivery settings, characteristics of the EBI, and respondents [5].

Sustainability is particularly important in the implementation of routine patient-reported outcome (PRO) surveillance and guideline-based symptom management interventions in cancer care settings [9–11]. PRO assessments capture patients' perspectives on their symptoms, functioning, and health-related quality of life, and can be used to monitor health status, target symptom-directed interventions, inform clinical decision-making, and facilitate patient-provider communication [12, 13]. Integrating PRO measures into routine oncology practice has been shown to reduce acute care visits and improve symptom control, patient satisfaction, and potentially overall survival [14–17]. However, implementing and sustaining PROs and cancer symptom management interventions can be challenging, as they require significant organizational commitment, infrastructure, resources, and engagement of multiple stakeholders [11, 18–20].

Despite the proliferation of sustainability frameworks and measures, several gaps remain in the assessment of sustainability capacity in clinical settings. Many existing tools lack consistent conceptual grounding, rely on lengthy item sets that are difficult to administer in time-limited healthcare environments, or have undergone limited psychometric testing outside of small or homogeneous samples [1, 3, 21]. Additionally, few measures are designed specifically to capture organizational capacity for sustaining evidence-based practices, a construct identified as central to long-term implementation success [7]. The CSAT was developed to address these limitations by providing a pragmatic, clinically oriented, and psychometrically robust measure of organizational sustainability capacity [22, 23].

The Clinical Sustainability Assessment Tool (CSAT) is an instrument for assessing the sustainability of evidence-based practices in healthcare settings [22]. The CSAT measures organizational capacity for sustainability across seven domains. Each domain is evaluated by 7 items; however, a recently validated short form of the CSAT (CSAT Short) reduced the number of items to 3 per domain [24]. Establishing shorter versions of surveys that maintain rigorous measurement increases pragmatism by reducing response burden and facilitating their use [25]. Among all measurement tools designed to capture sustainability in clinical settings, the CSAT has been shown to have the strongest measurement properties based on the Psychometric and Pragmatic Evidence Rating scale (PAPERS) criteria [26, 27]. However, further psychometric evaluation using modern psychometric methods, such as confirmatory factor analysis (CFA) and item response theory (IRT), is needed to establish the structural validity, measurement invariance, and item-level functioning of the CSAT Short.

This study sought to address several notable gaps in knowledge. First, it is important to replicate studies using established instruments to ensure their continued reliability and to provide additional validity evidence, particularly evidence of external validity. Additionally, to date, no invariance testing has been conducted using the CSAT Short to determine measurement equivalency across respondent roles, years in current role, and care delivery settings. Finally, the measurement properties of the CSAT have mostly been examined using classical test theory. Thus, item response theory analyses have not been previously conducted for the CSAT or CSAT Short. This study makes two novel contributions: it is the first to validate the CSAT Short across multiple research centers and staff roles, and the first to apply multidimensional item response theory (mIRT) to evaluate its psychometric performance. Cancer symptom management was selected as the test context because it represents a critical, multidisciplinary area in which implementation sustainability is particularly relevant, given the complexity of care coordination and the long-term need for integration into routine oncology practice.

The primary objectives of this study were to evaluate the extent to which the hypothesized factor structure of the CSAT Short provides a good fit to the data among a sample of clinical and non-clinical respondents across three Research Centers within the Improving the Management of symPtoms during And following Cancer Treatment (IMPACT) consortium [28]. Each of the trials [29–31] in the IMPACT consortium were hybrid type 2 effectiveness-implementation studies [32]. The overall goal of the IMPACT consortium is to conduct studies testing routine symptom surveillance and management interventions in ambulatory oncology care settings. We also conducted invariance testing to determine whether differences are observed by role, years of experience in current role, and between care delivery settings. Specifically, we conducted factor analyses to (a) determine internal consistency, (b) confirm the factor structure, and (c) test the measurement equivalency between respondent role (e.g., clinicians vs. non-clinicians), the number of years in current role (i.e., < 10 years or > 10 years), and across the three Research Centers. Additional objectives were to apply IRT to determine item-level functioning of the CSAT Short in diverse implementation contexts. This study followed the reporting guidelines for Confirmatory Factor Analysis checklist [33] to ensure comprehensive and transparent reporting included as Additional File 1.

## Methods

### Study design and setting

A cross-sectional survey design was used to assess healthcare personnel perceptions of the clinical sustainability of a symptom management intervention for cancer patients across diverse oncology outpatient practice settings. This study used data from the IMPACT Consortium, funded through the Cancer Moonshot^SM^ initiative, which is comprised of three Research Centers using hybrid effectiveness-implementation cluster randomized pragmatic trials to test integrated electronic systems to monitor and manage cancer symptoms, a Coordinating Center (CC) led by RTI International that provides scientific expertise and logistical support, and NCI program scientists. Research Centers included: 1) Northwestern University (NU); IMPACT Research Center; 2) Enhanced, EHR-facilitated cancer symptom control (E2C2) Research Center led by Mayo Clinic; and 3) the Symptom Management IMplementation of Patient Reported Outcomes in Oncology (SIMPRO) Research Center, a network of six health systems led by Dana-Farber Cancer Institute in Boston. These three hybrid type 2 effectiveness-implementation studies were conducted between 2019 and 2024 [29–31]. Despite the similarities between the three Research Centers and their shared overall goal of improving cancer symptom management, it is worth noting that there were minor distinctions between the symptom management interventions used across the three Research Centers. For detailed descriptions of these interventions, see the IMPACT overview paper [28] and the study protocol papers [29–31].

### Participants and data collection procedures

Data were collected 12 months after the transition from the control condition into the intervention condition at each of three Research Centers. The 12-month post-transition time point was selected to allow sufficient time for implementation processes and clinical workflows to stabilize, thereby capturing organizational sustainability capacity after initial adoption rather than during early implementation. Participants included clinical and non-clinical staff involved in implementing PROs and cancer symptom management interventions at participating clinics. Some staff had dual roles as administrators and clinicians. Eligible staff were identified by clinical leads at each site and invited via email to complete the CSAT Short. Because this study used an existing implementation network, response rates were not systematically tracked; however, participation rates were consistent with internal staff survey norms in similar studies of healthcare professionals. Although all staff were invited to participate, it is possible that individuals with greater interest in sustainability or implementation processes were more likely to respond. The survey was administered electronically using REDCap or Qualtrics. Survey administration platform selection reflected existing institutional infrastructure at each Research Center; however, survey content and item presentation were identical across REDCap [34] and Qualtrics [35], both of which are widely used and validated platforms for research data collection. Participants from the NU-IMPACT and SIMPRO Research Centers provided informed consent before completing the CSAT Short; at the E2C2 Research Center, the study was determined by the Institutional Review Board (IRB) to be exempt. Participants provided limited demographic information (role, years in current professional role, and Research Center site), answered questions about their level of involvement with the implementation, and completed a package of implementation surveys [36]. Additional sample demographic characteristics were not captured owing to concerns about possible respondent identifiability.

### Instrument: CSAT short form (CSAT Short)

The CSAT, an instrument designed to assess the sustainability of evidence-based practices in clinical settings, is available in multiple versions, including 49-item, 35-item, and 21-item versions [22]. In this study, we evaluated the 21-item CSAT Short (available at http://www.sustaintool.org/).^1^ The CSAT Short, like all CSAT versions, assesses seven key domains of sustainability with three items in each domain: *Engaged staff and leadership, Engaged stakeholders, Organizational readiness, Workflow integration, Implementation and training, Monitoring and evaluating,* and *Outcomes and effectiveness.* Items are rated on a 7-point Likert scale, with higher scores indicating more favorable responses regarding sustainability (1 = little or no extent, 7 = to a very great extent), and summative scores are calculated by domain and overall to assess the extent to which the service setting incorporates the contextual factors that promote sustainability. Example items include, “the practice is built into the clinical workflow” and “the practice is valued by a diverse set of interested parties”. Confirmatory Factor analysis of the CSAT Short has demonstrated high internal consistency (Cronbach’s alpha’s of 0.84–0.92 for each domain) [24].

### Analysis

Since the seven factor structure of the CSAT has been confirmed in prior studies [22], we sought to determine if the shortened 21 item CSAT maintained the same core underlying seven factor structure as the original instrument, using CFA. Weighted least squares mean and variances (WLSMVs) estimation with delta parameterization was used, given that the data are ordered-categorical scale items [37]. All analyses were conducted in M*plus* version 8.4 [38], which allows for WLSMV estimation when designating that the data are categorical and uses pairwise deletion for missing data. Bootstrapping for confidence intervals was not applied, as WLSMV provides robust standard errors, means, and variance adjusted χ2 statistics. The fit of each model was determined by examining fit indices including the χ2 statistic, the comparative fit index (*CFI* ≥ 0.95) [39], the Tucker-Lewis index (*TLI* ≥ 0.95) [40], the root mean square error of approximation (*RMSEA* ≤ 0.08) [41], and the standardized root mean square residual (*SRMR* ≤ 0.08) [42]. Because the CSAT Short is a previously validated instrument with a well-established seven-factor structure, we did not conduct and exploratory factor analysis prior to CFA [22]. We prioritized the unitary, second-order structure of the CSAT, given that it has been established as having a second-order structure with 7 latent, first-order factors.

Although rules of thumb for CFA suggest a minimum sample size of 100 to 200 participants, MacCallum et al. [43] indicate that it is more important to maintain communalities (i.e., the portion of the variance that is accounted for by the common factors, or R-square values) of at least 0.60 among factors. Therefore, we report communalities for each factor.

#### Scale reliability

Scale reliability was calculated using the Raykov reliability estimate, rho (*ρ*) [44], which is more appropriate for use in CFA than Cronbach’s coefficient *a*, and which has been shown to provide unreliable estimates of scale reliability [45–47]. Raykov’s rho (*ρ*) can be interpreted similarly to classic interpretations of reliability, as an indicator of precision representing the amount of true score variance divided by total variance.

#### Invariance testing

Because our sample included respondents from multiple projects, roles, and years of experience, we tested whether the CSAT Short functioned equivalently across these groups. Non-clinicians (i.e., hospital administrators and leaders, administrative and support staff, IT personnel) were compared with clinicians (oncologist and other physicians, nurse practitioners, physician assistants, pharmacist, social workers). There were individuals with dual roles (i.e., clinician and administrators) who were included in the non-clinician group for sample balancing purposes. We also tested for invariance between respondents with greater than or fewer than 10 years of experience in their current role, and across the three Research Centers. The sample breakdown of these categories by site are displayed in Table 1.

**Table 1:** Breakdown of role and year groupings by site for Invariance Testing

	**Role Group**		**Role Years**	
**Site**	**Clinicians**	**NonClinicians**	** < 10 years**	** > 10 years**
	***n* (%)**	***n* (%)**	***n* (%)**	***n* (%)**
**NU**	44 (24%)	24 (39%)	51 (35%)	17 (19%)
**SIMPRO**	121 (65%)	36 (58%)	84 (58%)	73 (79%)
**E2C2**	20 (11%)	2 (3%)	11 (8%)	2 (2%)
**Total**	185	62	146	92

To evaluate whether the CSAT Short performed equivalently across key subgroups, we first established configural invariance by testing the CFA model separately in each group to ensure that the same factor structure was acceptable across subgroups. Next, to assess equality of the parameters, we conducted the Langer-improved Wald test [48], which provides an omnibus test of parameter equivalence across groups and has been shown to approximate simultaneous tests of metric and scalar invariance [49]. This approach was selected because traditional stepwise multi-group CFA methods did not converge due to subgroup sample sizes and model complexity. The Wald Test was, therefore, used as a robust and efficient alternative that evaluates equality of all parameters simultaneously while maintain the confirmatory factor structure. If the Wald test is non-significant (*p* > 0.05), this suggests measurement non-invariance between groups (i.e., that the measurement model differs across the groups being compared, or that the groups interpret or responded differently to the items in the instrument).

#### Multidimensional IRT analyses

Multidimensional item response theory (mIRT) analyses were employed to evaluate the psychometric properties of the CSAT Short at the item level. Unlike unidimensional IRT, which assumes all items measure a single latent trait, mIRT allows for the simultaneous modeling of multiple latent dimensions via higher order factors [50]. Based on prior research supporting a 7-factor structure for the CSAT [22], a second-order mIRT model was specified in which each item loaded on one of seven specific factors, which in turn loaded on a general factor (G) representing their shared variance. Items 1–3 were specified to load on the *Engaged Staff & Leadership* factor, items 4–6 on Engaged Stakeholders, items 7–9 on *Organizational Readiness*, items 10–12 on *Workflow Integration*, items 13–15 on *Implementation & Training*, items 16–18 on *Monitoring & Evaluation*, and items 19–21 on *Outcomes & Effectiveness*.

The model item parameters, including discrimination and difficulty, were estimated using the Metropolis–Hastings Robbins-Monro (MH-RM) algorithm, a Markov chain Monte Carlo (MCMC) method, as implemented in the mIRT package for R [51]. Discrimination parameters represent the strength of the relationship between an item and the latent trait, while difficulty parameters represent the location of the item on the latent trait required for a 50% probability of endorsement. A quasi-Monte Carlo integration method with 1,000 integration points was used to approximate the likelihood function. The model was set to run for up to 5,000 cycles to ensure convergence.

Overall model fit was evaluated using the *M2* statistic and the same fit indices and cut-off criteria described above for the CFA models, including *RMSEA*, *SRMR*, *CFI*, and *TLI*. In addition to assessing the overall fit, item-level fit was assessed using the *S-X*^*2*^ statistic, which compares observed and expected response frequencies for each item using a chi-square test [52, 53]. A non-significant *S-X*^*2*^ (*p* > 0.05) indicates good item fit [53]. The mIRT implementation of *S-X*^*2*^ also computes an *RMSEA*.*S-X*^*2*^, *RMSEA.S-X*^*2*^ an index of item misfit based on the *S-X*^*2*^ statistic, assesses the discrepancy between the observed and expected response patterns for each item, accounting for model complexity. Lower values of *RMSEA.S-X*^*2*^ indicate better item fit, with values below 0.05, between 0.05 and 0.08, between 0.08 and 0.10, and above 0.10 indicating good, acceptable, marginal, and poor fit, respectively [54].

## Results

### Descriptive statistics

The analytic sample consisted of 256 members of the patient care and administrative staff drawn from three Research Centers participating in the IMPACT Consortium: 1) SIMPRO (*n* = 166; 64.84% response rate); 2) NU IMPACT (*n* = 68; 26.56% response rate), and E2C2 (*n* = 22; 8.59% response rate). Most participants were categorized in the clinician group (*n* = 185, 74.90%), with 62 participants (25.10%) comprising the non-clinician group. Regarding years in their current role, 146 participants (61.34%) had less than 10 years in current role, while 92 participants (38.66%) had 10 or more years in current role. As previously noted, additional sample demographic characteristics were not captured owing to concerns about respondent identifiability. CSAT items and response frequencies are displayed in Table 2.

**Table 2:** Frequency Distributions of Responses to CSAT Short Form Items

		**Item Responses**						
**CSAT Short Item**	**Item Text**	**1** *N*(%)	**2** *N*(%)	**3** *N*(%)	**4** *N*(%)	**5** *N*(%)	**6** *N*(%)	**7** *N*(%)
1	The practice has engaged, ongoing champions	18(8%)	11(5%)	19(9%)	36(17%)	34(16%)	49(23%)	47(22%)
2	The practice has a leadership team made of multiprofessional partnerships	15(7%)	7(3%)	14(7%)	25(12%)	39(19%)	47(22%)	63(30%)
3	The practice has team-based collaboration and infrastructure	17(8%)	8(4%)	17(8%)	28(13%)	39(19%)	49(23%	51(24%)
4	There is respect for all stakeholders involved in the practice	13(6%)	12(6%)	11(5%)	25(12%)	26(13%)	54(27%)	62(31%)
5	The practice is valued by a diverse set of stakeholders	14(7%)	10(5%)	18(9%)	30(16%)	36(19%)	43(22%)	42(22%)
6	The practice engages other medical teams and community partnerships as appropriate	20(11%)	7(4%)	14(7%)	33(18%)	31(17%)	49(26%)	34(18%)
7	The practice is well integrated into the operations of the organization†	14(8%)	14(8%)	23(13%)	30(17%)	30(17%)	47(26%)	20(11%)
8	The practice has feasible and sufficient resources to achieve its goals	17(9%)	21(11%)	25(16%)	31(16%)	43(22%)	39(20%)	21(11%)
9	The practice has adequate staff to achieve its goals	16(7%)	27(12%)	32(14%)	39(18%)	38(17%)	45(20%)	26(12%)
10	The practice is built into the clinical workflow	20(8%)	25(10%)	24(10%)	39(16%)	48(20%)	52(22%)	34(14%)
11	The practice is easy for clinicians to use	16(7%)	22(10%)	19(8%)	39(17%)	41(18%)	47(18%)	42(19%)
12	The practice integrates well with established clinical practices	18(8%)	32(14%)	24(10%)	35(15%)	35(15%)	50(22%)	37(16%)
13	The reason for the practice is clearly communicated to and understood by all staff	15(6%)	13(6%)	27(12%)	42(18%)	45(19%)	47(20%)	44(19%)
14	Staff receive ongoing coaching, feedback, and training	36(16%)	24(11%)	22(10%)	37(16%)	35(15%)	45(20%)	29(13%)
15	Practice implementation is guided by feedback from stakeholders†	21(11%)	15(8%)	15(8%)	29(15%)	39(21%)	36(19%)	35(18%)
16	The practice has measurable process components, outcomes, and metrics	15(7%)	14(7%)	8(4%)	28(14%)	37(18%)	48(24%)	52(26%)
17	Evaluation and monitoring of the practice are reviewed on a consistent basis	14(7%)	11(6%)	13(7%)	24(12%)	31(16%)	52(27%)	51(26%)
18	Practice monitoring, evaluation, and outcomes data are routinely reported to the clinical care team	25(12%)	14(7%)	17(8%)	24(12%)	31(15%)	49(24%)	48(23%)
19	The practice has evidence of beneficial outcomes	19(10%)	8(4%)	11(6%)	32(17%)	38(20%)	39(21%)	41(22%)
20	The practice is cost-effective†	18(10%)	12(6%)	14(8%)	30(16%)	32(17%)	41(22%)	40(21%)
21	The practice is clearly linked to positive health or clinical outcomes	16(11%)	5(3%)	6(4%)	24(17%)	25(17%)	40(28%)	29(20%)

^†^ item language was adapted in the final published version of the CSAT Short

### CFA model building

**Fig. 1:**
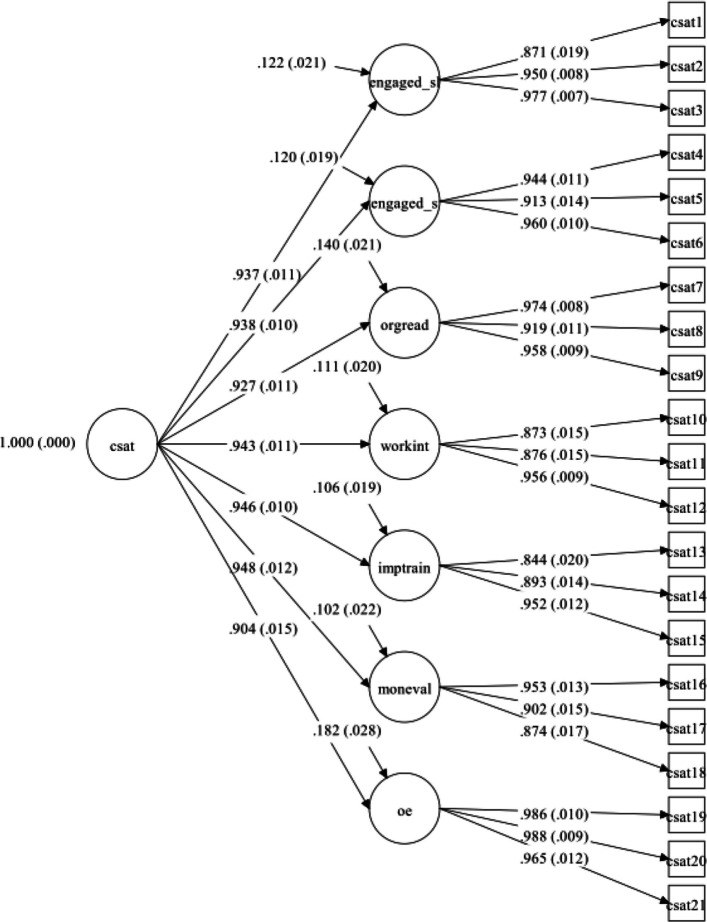
CSAT Short Form 2nd Order CFA Factor Structure (Standardized). N = 256, CFI =.99, TLI =.98, *X*^2^(182) = 658.99, *p* <.001; SRMR =.031, RMSEA =.1

The first CFA model (H_0_) included no cross-loadings or correlated error terms. All 21 items were included, with three items loading onto each of the seven subscales, and all subscales loading onto one unitary, second-order factor. Marker indicators were specified as the first item of each subscale. Prior to the CFA analysis, the data were evaluated for univariate and multivariate outliers by examining leverage indices for each participant. No univariate or multivariate outliers were detected. The model was overidentified with 182 *df*, and there were 131 missing data patterns in the retained model. Global model fit indices indicated excellent fit, (*N* = 256, *CFI* = 0.99, *TLI* = 0.98, *X*^2^(182) = 658.99, *p* < 0.001; *SRMR* = 0.031, *RMSEA* = 0.10). However, the *RMSEA* value suggested that the model could be re-specified to improve fit and be made more parsimonious. Therefore, localized areas of ill-fit were explored, beginning with modification indices. These indicated that improvements could be made by correlating the error terms of both the Engagement factors (H_1_) as well as between the Organizational Readiness and Monitoring and Evaluating factors (H_2_). Chi-square tests were conducted between the models separately (i.e., H_0_ vs. H_1_; H_0_ vs. H_2_) as well as correlating error terms for both factors together in the same model (H_0_ vs. H_3_); however, all restricted models significantly worsened fit compared to H_0_, despite each slightly improving RMSEA values. Therefore, the H_0_ model was retained. All freely estimated unstandardized parameters were statistically significant (*p-*values < 0.001). Figure 1 displays the factor structure and includes the standardized factor loading and error terms. Although factor loadings were high (0.84–0.99), suggesting strong item–factor relationships, this may also reflect shared variance due to the common administration context rather than overfitting or method bias. Table 3 includes the unstandardized and standardized factor loadings, composite reliability, and R^2^ values for the final model. Factor loading estimates revealed that the indicators were strongly related to their ostensible latent factors (range of *R*^*2*^*s* = 0.71–0.98).

**Table 3:** CFA of the CSAT Short Form with Reliability and *R*^*2*^

Construct/Item	Unstandardized factor loading (SE)	Standardized factor loading (SE)	Composite Reliability *ρ*	*R*^*2*^
CSAT	–	–	**.93**	–
Engaged staff and leadership	**1.00 (.00)**	**.94 (.02)**	**.93**	**.88**
CSAT1	1.00 (.00)	.87 (.02)		.76
CSAT2	1.09 (.02)	.95 (.01)		.90
CSAT 3	1.12 (.02)	.98 (.01)		.96
Engaged stakeholders	**1.09 (.03)**	**.94 (.01)**	**.95**	**.88**
CSAT4	1.00 (.00)	.94 (.01)		.89
CSAT5	.97 (.02)	.92 (.01)		.83
CSAT6	1.02 (.01)	.96 (.01)		.92
Organizational readiness	**1.11 (.03)**	**.93 (.01)**	**.90**	**.86**
CSAT7	1.00 (.00)	.97 (.01)		.95
CSAT8	.94 (.01)	.92 (.01)		.85
CSAT9	.98 (.01)	.96 (.01)		.92
Workflow integration	**1.01 (.03)**	**.94 (.03)**	**.93**	**.89**
CSAT10	1.00 (.00)	.87 (.02)		.76
CSAT11	1.00 (.02)	.88 (.02)		.77
CSAT 12	1.10 (.02)	.96 (.01)		.91
Implementation and training	**.978 (.03)**	**.95 (.01)**	**.89**	**.89**
CSAT13	1.00 (.00)	.84 (.02)		.71
CSAT14	1.06 (.03)	.89 (.01)		.80
CSAT15	1.13 (.03)	.95 (.01)		.91
Monitoring and evaluating	**1.11 (.03)**	**.95 (.02)**	**.91**	**.90**
CSAT16	1.00 (.00)	.95 (.01)		.91
CSAT17	.95 (.02)	.90 (.01)		.81
CSAT18	.91 (.02)	.87 (.02)		.76
Outcomes and effectiveness	**1.09 (.03)**	**.90 (.02)**	**.95**	**.82**
CSAT19	1.00 (.00)	.99 (.01)		.97
CSAT20	1.00 (.01)	.99 (.01)		.98
CSAT21	.98 (.02)	.97 (.01)		.93

*N* = 225*, CFI* =.95, *TLI* =.94, *X*^*2*^(182) = 858.9, *p* <.001; *SRMR* =.048, *RMSEA* =.13

*Note: *bolded text indicates full scale or subscale level values

Composite reliability across all factors ranged from 0.89–0.953. Generally, reliability coefficients of 0.90 and above are considered “excellent” and above 0.80 are “very good” [55]. *R*^*2*^ results (i.e., communalities of each item) indicated our sample size was adequate (range: 0.71–0.98). Polychoric correlations between all items and subscales are presented in Table 4. The retained sample (*N* = 256) exceeded conventional minimum recommendations for CFA, which typically requires 100–200 participants depending on communalities and model complexity [43]. Our 7-factor model included 21 items, yielding an item-to-factor ratio of 3:1. While this ratio is modest, the high communalities (≥ 0.60) and strong factor loadings observed support stable parameter estimation [56].

**Table 4:** Polychoric Correlations Between CSAT Short Form Items

	1	2	3	4	5	6	7	8	9	10	11	12	13	14	15	16	17	18	19	20	21
1	–	–	–	–	–	–	–	–	–	–	–	–	–	–	–	–	–	–	–	–	–
2	.71	–	–	–	–	–	–	–	–	–	–	–	–	–	–	–	–	–	–	–	–
3	.72	.94	–	–	–	–	–	–	–	–	–	–	–	–	–	–	–	–	–	–	–
4	.57	.70	.70	–	–	–	–	–	–	–	–	–	–	–	–	–	–	–	–	–	–
5	.52	.71	.70	.85	–	–	–	–	–	–	–	–	–	–	–	–	–	–	–	–	–
6	.49	.70	.71	.83	.93	–	–	–	–	–	–	–	–	–	–	–	–	–	–	–	–
7	.49	.65	.65	.66	.68	.69	–		–	–	–	–	–	–	–	–	–	–	–	–	–
8	.48	.64	.62	.60	.60	.62	.81	–	–	–	–	–	–	–	–	–	–	–	–	–	–
9	.61	.63	.65	.61	.63	.62	.67	.75	–	–	–	–	–	–	–	–	–	–	–	–	–
10	.48	.43	.48	.47	.42	.44	.52	.51	.66	–	–	–	–	–	–	–	–	–	–	–	–
11	.39	.39	.40	.40	.38	.41	.52	.54	.57	.73	–	–	–	–	–	–	–	–	–	–	–
12	.50	.46	.53	.52	.43	.51	.57	.56	.65	.75	.87	–	–	–	–	–	–	–	–	–	–
13	.59	.56	.61	.48	.44	.53	.60	.55	.63	.67	.63	.69	–	–	–	–	–	–	–	–	–
14	.52	.65	.67	.55	.48	.56	.56	.65	.64	.58	.50	.58	.76	–	–	–	–	–	–	–	–
15	.44	.68	.65	.67	.65	.70	.68	.64	.58	.45	.48	.54	.63	.74	–	–	–	–	–	–	–
16	.59	.60	.65	.56	.51	.61	.54	.49	.55	.58	.54	.62	.65	.62	.64	–	–	–	–	–	–
17	.53	.57	.59	.59	.56	.63	.54	.55	.50	.60	.57	.61	.61	.62	.68	.74	–	–	–	–	–
18	.58	.54	.57	.56	.47	.53	.45	.47	.55	.54	.55	.61	.63	.71	.60	.71	.82	–	–	–	–
19	.48	.56	.58	.54	.58	.63	.53	.59	.55	.42	.55	.56	.51	.54	.59	.69	.65	.62	–	–	–
20	.49	.56	.57	.50	.57	.63	.56	.60	.56	.38	.54	.56	.57	.57	.61	.67	.64	.59	.93	–	–
21	.47	.52	.56	.57	.58	.65	.62	.59	.49	.34	.42	.51	.44	.43	.64	.62	.59	.49	.83	.83	–

### Measurement invariance

Results of measurement invariance across the three Research Centers, role groups (clinician vs. non-clinician) and years in current role (≤ 10 years vs. > 10 years) are displayed in Table 5. For clinician vs. non-clinician respondents, when CFAs were run separately on each group, both clinicians and non-clinicians had statistically significant factor loadings (*p* < 0.001) that were salient, with standardized loadings ranging from 0.68 to 0.93 for clinicians and 0.71 to 0.99 for non-clinicians. The non-significant Wald test (*Wald*(8) = 6.27, *p* = 0.51) supported metric and scalar invariance between the two groups. Similarly for years in current role, when CFAs were run separately, both groups (< 10 years and ≥ 10 years) had statistically significant and salient factor loadings, with standardized estimates ranging from 0.80 to 0.98 for those with less than 10 years in current role and 0.78 to 0.96 for those with 10 or more years. The non-significant Wald test (*Wald*(7) = 7.93, *p* = 0.34) supports measurement invariance between groups with 10 or more years and those with less than 10 years in current role. Finally, the significant Wald test (*Wald*(6) = 18.05, *p* = 0.012) did not support measurement invariance across the three Research Centers indicating differences in responding depending on which Research Center they were associated with.

**Table 5:** Tests of Measurement Invariance

	*X*^*2*^	*df*	*RMSEA* (90%CI)	*SRMR*	*CFI*	*TLI*	*Wald* Value	*df*	*P*-value
*Research Center Grouping*									
Single Group Solutions									
NU (*n* = 68)	356.1***	182	.12 (.10–.14)	.06	.98	.98	–	–	–
SIMPRO (*n* = 166)	476.8***	182	.11 (.09–.141	.03	.99	.99	–	–	–
E2C2 (*n* = 22)	222.7*	182	.10 (.04–.14)	.13	.99	.99	–	–	–
Measurement Invariance									
Wald Test	787.7***	438	.08 (.07–.09)	.04	.99	.99	18.05*	6	.012
*Years in role Grouping*									
Single Group Solutions									
< 10 (*n* = 146)	412.9***	182	.09 (.08–.11)	.03	.99	.99	–	–	–
≥ 10 (*n* = 92)	463.5***	182	.13 (.12–.14)	.06	.98	.97	–	–	–
Measurement Invariance									
Wald Test	862.6***	444	.09 (.08–.10)	.05	.99	.99	7.93	7	.34
*Role Grouping*									
Single Group Solutions									
Clinicians (*n* = 185)	560.8***	182	.11 (.09–.12)	.03	.99	.98	–	–	–
Non-Clinicians (*n* = 62)	598.3***	182	.10 (.08–.12)	.06	.97	.97	–	–	–
Measurement Invariance									
Wald Test	841.9***	435	.09 (.08–.10)	.04	.99	.99	6.27	7	.51

*RMSEA* root mean square error of approximation, *90% CI* 90% confidence interval for RMSEA, *SRMR* standardized root mean square residual, *CFI* Comparative Fit Index, *TLI* Tucker–Lewis Index

^*^ = *p* <.05, ** = *p* <.01, *** = *p* <.001

### Multidimensional IRT

The second-order mIRT model demonstrated acceptable fit based on most indices (*M2*(56) = 148.69, *p* < 0.001; *RMSEA* = 0.059, 90% CI [0.048,0.071]; *SRMSR* = 0.057; *CFI* = 0.917), though the *TLI* (0.845) was slightly below the recommended threshold. Factor correlations among the seven CSAT domains and the general factor (*G*) were high, ranging from 0.798 to 1.000 (Table 6).

**Table 6:** MIRT Factor Correlations Among the Seven CSAT Short Form Domains and the General Factor

**Factor**	**2nd order General factor**	**Engaged Staff & Leadership**	**Engaged Stakeholders**	**Organizational Readiness**	**Workflow Integration**	**Implementation & Training**	**Monitoring & Evaluation**	**Outcomes & Effectiveness**
2nd order General factor	1							
Engaged Staff & Leadership	0.975	1						
Engaged Stakeholders	1	0.97	1					
Organizational Readiness	0.937	0.916	0.936	1				
Workflow Integration	0.907	0.859	0.909	0.989	1			
Implementation & Training	0.971	0.979	0.967	0.964	0.923	1		
Monitoring & Evaluation	0.933	0.934	0.93	0.908	0.883	0.959	1	
Outcomes & Effectiveness	0.814	0.806	0.812	0.814	0.798	0.833	0.848	1

Item-level fit was assessed using the *S-X*^*2*^ statistic and its associated *p*-value (*p. S-X*^*2*^), as well as the *RMSEA*.*S-X*^*2*^index (Table 7). All items had significant *S-X*^*2*^ values (*p*. *S-X*^*2*^ < 0.001), suggesting poor fit; however, this statistic is highly sensitive to sample size and does not necessarily indicate poor practical fit. The corresponding *RMSEA*.*S-X*^*2*^ values ranged from 0.066 to 0.125. Six items (*Engaged Champions, Diverse Value, Ease of Use, Clear Communication, Stakeholder Feedback, and Data Reporting*) had *RMSEA.S-X*^*2*^ values between 0.05 and 0.08, indicating acceptable fit. Nine items (*Sufficient Resources, Adequate Staff, Organizational Integration, Workflow Fit, Clinical Practice Fit, Ongoing Training, Consistent Evaluation, Beneficial Evidence, and Cost Effective*) had *RMSEA.S-X*^*2*^ values between 0.08 and 0.10, suggesting marginal fit. Five items (*Leadership Team, Team Collaboration, Stakeholder Respect, Engage Partners, and Measurable Outcome*) exhibited *RMSEA.S-X*^*2*^ values above 0.10, indicating poor fit.

**Table 7:** Item-Level Fit Statistics or the Second-Order Multidimensional Item Response Theory (MIRT) Model of the CSAT

**Factor Name**	**Item Short Name**	**Factor Loading**	**Communality**	***S-X***^***2***^	***RMSEA***	***p*-value (*S-**** X*^*2*^**)**
Engaged Staff & Leadership	Engaged Champions	0.866	0.750	64.41	0.066	< 0.05
Engaged Staff & Leadership	Leadership Team	0.935	0.873	66.194	0.125	< 0.05
Engaged Staff & Leadership	Team Collaboration	0.955	0.912	58.639	0.125	< 0.05
Engaged Stakeholders	Stakeholder Respect	0.906	0.821	49.013	0.105	< 0.05
Engaged Stakeholders	Diverse Value	0.899	0.808	56.841	0.071	< 0.05
Engaged Stakeholders	Engage Partners	0.920	0.846	72.055	0.090	< 0.05
Organizational Readiness	Sufficient Resources	0.885	0.784	49.073	0.086	< 0.05
Organizational Readiness	Adequate Staff	0.877	0.768	95.754	0.099	< 0.05
Organizational Readiness	Organizational Integration	0.938	0.879	89.362	0.092	< 0.05
Workflow Integration	Workflow Fit	0.879	0.772	122.657	0.094	< 0.05
Workflow Integration	Ease Of Use	0.880	0.774	88.416	0.076	< 0.05
Workflow Integration	Clinical Practice Fit	0.940	0.884	96.724	0.085	< 0.05
Implementation & Training	Clear Communication	0.851	0.724	74.962	0.069	< 0.05
Implementation & Training	Ongoing Training	0.866	0.751	77.862	0.087	< 0.05
Implementation & Training	Stakeholder Feedback	0.891	0.795	58.371	0.066	< 0.05
Monitoring & Evaluation	Measurable Outcome	0.923	0.851	77.594	0.120	< 0.05
Monitoring & Evaluation	Consistent Evaluation	0.914	0.836	45.881	0.082	< 0.05
Monitoring & Evaluation	Data Reporting	0.886	0.786	67.451	0.074	< 0.05
Outcomes & Effectiveness	Beneficial Evidence	0.984	0.968	63.57	0.080	< 0.05
Outcomes & Effectiveness	Positive Link	0.988	0.976	57.18	0.068	< 0.05
Outcomes & Effectiveness	Cost Effective	0.897	0.804	52.144	0.101	< 0.05

Factor Name: This column lists the names of the latent factors in the MIRT model. Item Short Name: This column contains short, descriptive labels or numbers for each item in the CSAT. Factor Loading: Factor loadings represent the strength and direction of the relationship between each item and its corresponding latent factor. Factor loadings can range from −1 to 1, with values closer to 0 indicating weaker relationships. Communality: Communality (h2) is the proportion of an item's variance that is explained by the latent factors in the MIRT model. Communality values range from 0 to 1, with higher values indicating that a larger proportion of the item's variance is accounted for by the latent factors. S-X^2^: The S-X2 statistic is an item-level fit index that assesses the discrepancy between the observed and expected response patterns for each item. A non-significant S-X^2^ value (*p* > 0.05) indicates that the item fits the MIRT model well. RMSEA: The Root Mean Square Error of Approximation (RMSEA) is an index of item misfit based on the S-X^2^ statistic. It assesses the discrepancy between the observed and expected response patterns for each item, while accounting for the model's complexity. Lower values of RMSEA indicate better item fit. RMSEA values below 0.05 indicate good fit, values between 0.05 and 0.08 indicate acceptable fit, values between 0.08 and 0.10 indicate marginal fit, and values above 0.10 indicate poor fit

The discrimination parameters (a) for the items ranged from 2.758 to 10.804, indicating a strong relationship between the items and their respective latent factors (Table 8). The difficulty parameters (d) ranged from –14.315 to 18.791, suggesting that the items covered a wide range of the latent trait continuum.

**Table 8:** Parameter Estimates for the CSAT Items from the Second-Order Multidimensional Item Response Theory (MIRT) Model

Item Short Name	a1	a2	a3	a4	a5	a6	a7	a8	d1	d2	d3	d4	d5	d6
Engaged Champions	0	2.94	0	0	0	0	0	0	6.06	5.13	4.11	2.93	−1.97	−3.77
Leadership Team	0	4.47	0	0	0	0	0	0	8.98	7.82	6.34	4.79	−2.22	−4.24
Team Collaboration	0	5.46	0	0	0	0	0	0	10.24	9.01	7.20	5.29	−2.90	−5.79
Stakeholder Respect	0	0	3.64	0	0	0	0	0	7.85	6.20	5.24	4.00	−1.74	−3.69
Diverse Value	0	0	3.49	0	0	0	0	0	7.40	6.15	4.8	3.62	−2.55	−4.60
Engage Partners	0	0	3.99	0	0	0	0	0	7.44	6.65	5.47	3.93	−2.84	−5.68
Sufficient Resources	0	0	0	3.24	0	0	0	0	6.93	5.40	4.11	3.13	−3.13	−5.92
Adequate Staff	0	0	0	3.09	0	0	0	0	6.32	4.65	3.59	−1.94	−3.32	−5.68
Organizational Integration	0	0	0	4.59	0	0	0	0	8.75	6.03	4.35	−2.19	−3.94	−7.26
Workflow Fit	0	0	0	0	3.13	0	0	0	6.03	4.35	3.40	−1.27	−2.53	−4.74
Ease Of Use	0	0	0	0	3.14	0	0	0	6.41	4.72	3.89	2.73	−2.43	−4.34
Clinical Practice Fit	0	0	0	0	4.70	0	0	0	8.58	5.78	4.49	−2.01	−3.54	−6.38
Clear Communication	0	0	0	0	0	2.75	0	0	5.97	4.81	3.52	2.40	−2.02	−3.75
Ongoing Training	0	0	0	0	0	2.95	0	0	4.73	3.75	3.04	−1.62	−2.63	−4.79
Stakeholder Feedback	0	0	0	0	0	3.34	0	0	6.07	4.96	4.24	3.27	−2.90	−4.89
Measurable Outcome	0	0	0	0	0	0	4.06	0	8.18	6.51	5.82	4.30	−2.51	−4.63
Consistent Evaluation	0	0	0	0	0	0	3.84	0	7.87	6.52	5.44	4.15	−2.32	−4.45
Data Reporting	0	0	0	0	0	0	3.25	0	5.85	5.00	4.21	3.36	−2.17	−4.12
Beneficial Evidence	0	0	0	0	0	0	0	9.29	15.972	13.857	11.762	8.526	−6.978	−12.18
Positive Link	0	0	0	0	0	0	0	10.80	18.791	15.249	12.714	9.577	−7.994	−14.31
Cost Effective	0	0	0	0	0	0	0	3.44	6.731	6.1	5.541	4.279	−3.415	−5.63

a1-a8: These columns represent the discrimination parameters for each item on the eight dimensions (a1 to a8) in the MIRT model. Discrimination parameters indicate the strength of the relationship between an item and the corresponding dimension. Higher values suggest that the item is better at distinguishing between individuals with different levels of the latent trait on that specific dimension. A value of 0 indicates that the item does not load on that dimension

d1-d6: These columns represent the difficulty parameters for each item on the six response categories (d1 to d6) in the MIRT model. Difficulty parameters, also known as threshold parameters, indicate the level of the latent trait required for an individual to endorse a particular response category. In this case, there are six response categories for each item. The difficulty parameters are ordered, with d1 representing the threshold between the lowest response category and the next one, and d6 representing the threshold between the second highest and the highest response category. Negative values indicate that the response category is easier to endorse, while positive values suggest that a higher level of the latent trait is required to endorse that category

## Discussion

We comprehensively evaluated the measurement properties of the CSAT Short in three hybrid effectiveness-implementation trials evaluating cancer symptom surveillance and management interventions in routine cancer care settings. Results provide strong support for the CSAT Short’s multidimensional structure, reliability, and item-level functioning. Measurement invariance testing indicated that the CSAT Short functioned equivalently across respondent roles and years in current role, supporting meaningful within-setting subgroup comparisons. However, evidence of non-invariance across Research Centers suggests that contextual factors may influence how sustainability constructs are perceived and reported. Accordingly, findings support the use of the CSAT Short for assessing sustainability capacity within similar oncology implementation settings, while cautioning against unqualified comparisons across healthcare systems or organizational contexts.

CFA results indicated that the seven-factor structure of the CSAT Short, as proposed by Malone et al. [22] provided good fit to our data. The strong factor loadings and high composite reliability estimates observed in our study further demonstrate the robustness of the CSAT Short's factor structure and its ability to reliably measure the key domains of sustainability. High inter-factor correlations suggest that, although the subscales measure related constructs, the strong higher-order factor structure supports use of both subscale and total scores, depending on analytic or implementation needs. Measurement invariance testing revealed that the CSAT Short exhibited configural, metric, and scalar invariance across respondent roles (clinicians vs. non-clinicians) and years in current role (< 10 years vs. ≥ 10 years). Although some items showed suboptimal fit in mIRT, the overall model demonstrated strong fit and reliability. These findings suggest that the CSAT Short measures sustainability in a consistent manner across these subgroups, allowing for meaningful comparisons of CSAT Short scores between clinicians and non-clinicians, as well as between those with varying levels of experience in their current role. This is an important contribution to the literature, as previous studies have not examined the measurement invariance of either the CSAT or the CSAT Short [22, 24]. However, we found evidence of variance across Research Centers, indicating that the CSAT Short's factor structure may differ depending on the specific healthcare setting, effecting the validity of comparing scores between healthcare settings; however, this effect may also be a consequence of this study’s relatively small sample size. Still, this finding underscores the importance of considering contextual factors when interpreting CSAT Short scores and highlights the need for further research to explore this across settings and to isolate potential sources of this variability. Moreover, the observed lack of observed invariance across Research Centers may reflect contextual differences in implementation processes, organizational culture, or local adaptations of the intervention. Future studies should employ multilevel or hierarchical modeling to disentangle organizational-level from individual-level sources of variance.

The mIRT analysis provided additional insights into the item-level functioning of the CSAT Short, supporting the hierarchical structure of the scale with seven first-order factors and one second-order general factor. The discrimination parameters from the mIRT analysis were high, indicating that the items effectively differentiate individuals with varying levels of sustainability. The wide range of difficulty parameters suggests that the CSAT Short items also cover a broad spectrum of the latent trait continuum. Taken together, these findings support the precision and sensitivity of the CSAT Short in measuring different levels of perceived intervention sustainability. However, item-level fit statistics revealed that several items exhibited marginal to poor fit. These results should be interpreted cautiously, as the *S-X*^*2*^ statistic is exceptionally sensitive to sample size and may indicate misfit even for well-fitting items in large samples [57, 58]. Future studies are needed to further investigate item fit and to determine the need for scale refinement.

This study provides practical guidance for researchers, practitioners, and policymakers seeking to assess sustainability capacity within oncology-based hybrid effectiveness–implementation studies. Because the CSAT Short demonstrated strong reliability and measurement consistency across groups, it can be used confidently to assess and compare sustainability within healthcare settings similar to those in the IMPACT consortium. The CSAT Short helps identify which sustainability domains need improvement, enabling organizations to design targeted strategies for long-term success. For example, if a healthcare organization scores low on the Workflow Integration domain, interventions focused on optimizing the integration of cancer symptom surveillance and management interventions into existing clinical workflows can be implemented. Such targeted strategies can ultimately contribute to the improvement of patient care and outcomes in cancer settings. Moreover, given that the CSAT Short exhibits strong psychometric properties while eliminating 28 items from the full-scale CSAT, the CSAT Short can also reduce response burden among participants and provide a more efficient, parsimonious assessment of sustainability.

Several caveats should be considered in interpreting our findings. Although the sample size was adequate based on communalities, larger samples are preferable in mIRT to ensure accurate parameter estimates [59]. Sample size also limited our ability to conduct more robust, step-wise multiple group CFAs, such as conducting chi-square tests of equal form (i.e., configural invariance), followed by testing equal factor loadings and equal thresholds (i.e., scalar and metric invariance) [60]. We were unable to achieve model convergence with these tests, and instead employed the Langer-improved Wald test [48], which simultaneously tests equality constraints on loadings and intercepts across groups and is statistically analogous to combined metric and scalar invariance testing. This approach provided an assessment of measurement equivalence without requiring large subgroup samples. Measurement non-invariance across Research Centers limits the interpretation of between-site comparisons and underscores the need for additional cross-context validation. Thus, future studies with larger samples can extend these analyses using full stepwise multi-group CFA framework to identify which specific levels of invariance contribute to observed differences across Research Centers.

The timing of data collection may also have influenced the observed psychometric properties of the CSAT Short. Assessing sustainability capacity 12 months after transition likely reflects more established implementation conditions, which may strengthen internal consistency and factor structure estimates. At the same time, this timing may accentuate contextual differences across healthcare systems, potentially contributing to the observed lack of measurement invariance across Research Centers.

The responses to the CSAT in the IMPACT consortium might also be influenced by the nature of the studies themselves. All three trials were hybrid type 2 effectiveness-implementation studies, and while the level of pragmatism of the strategies is not a 1:1 correspondence to the type of hybrid [61], the indications for a hybrid type 2 study are stated as such in the most recent guidance: “a type 2 necessitates deployment of implementation strategies developed and hypothesized to be feasible and impactful in real-word settings (a clear distinction from type 1)” [32]. Type 2 studies might explicitly evaluate the feasibility of the strategies while a type 3 requires that the strategies have established feasibility from prior trials/studies [62]. Thus, the results of this study on the CSAT might be most relevant to hybrid types 2 and 3 and may not hold in hybrid type 1 studies where implementation strategies are not necessarily (and are in fact unlikely to be) pragmatic and feasible for use at scale. This is an area for future validation and comparison between hybrid types.

The relatively small sample for the E2C2 site (*n* = 22) may have contributed to model instability and should be addressed in future replication studies. Future research should validate the CSAT Short in non-cancer and non–patient-reported outcome intervention contexts to further establish generalizability. The CFA *RMSEA* of 0.10, slightly exceeding the conventional threshold (< 0.08), likely reflects the model’s complexity and modest subgroup sizes rather than substantive misfit. Given the robust loadings and consistent fit across subgroups, this value does not meaningfully detract from the model’s validity. The slightly lower *TLI* (0.845) in the mIRT analysis similarly likely reflects model complexity and strong item discrimination parameters rather than poor fit. Although a differential item functioning (DIF) analysis was considered, subgroup sample sizes were insufficient for stable estimation; this remains an important direction for future research. Because we did not collect demographic data from our participants due to concerns about respondent identifiability, we were unable to test invariance based on factors such as age, gender, or race/ethnicity.

Furthermore, our study focused on the sustainability of routine symptom surveillance and management in cancer care settings, and thus the generalizability of our findings to other healthcare delivery contexts remains to be established. We did not examine the predictive validity of the CSAT Short or its relationship with other sustainability outcomes, such as the long-term effectiveness or the cost-effectiveness of routine surveillance with PRO systems. Although we sought to replicate the 7-factor structure established by CSAT developers, evidence from the mIRT indicated high factor correlations between the seven CSAT domains and the second-order general factor. This suggests that the factor structure may be better represented by a single factor; future research should explore this observation. At the same time, the strengths of our study include our use of a combination of classical and modern psychometric methods, and a sample drawn from across diverse cancer care delivery settings. Because participants self-selected into the study, and the response rates were relatively low at two Research Centers (NU IMPACT = 26.56%, E2C2 = 8.59%), the sample may overrepresent individuals who are more engaged in implementation or sustainability-related work or is otherwise not representative, which could limit generalizability to the broader population of clinical and administrative staff. We did not conduct a formal Monte Carlo or sensitivity analysis due to the model’s parsimony and observed empirical adequacy, but future validation studies with larger or more diverse samples could extend this evaluation using simulation-based methods to confirm the robustness of parameter stability.

## Conclusions

This study makes a unique contribution to the literature on sustainability assessment and implementation science in oncology-based hybrid type 2 and 3 effectiveness-implementation studies and among healthcare personnel with varying roles and levels of experience. We demonstrated the CSAT Short’s strong reliability and structural validity, and our findings advance the evidence base relative to its multidimensional structure and its potential to promote a more parsimonious assessment of the long-term integration of evidence-based practices. However, the lack of measurement invariance across Research Centers indicates that CSAT Short scores should be interpreted cautiously when making comparisons across healthcare systems or organizational contexts. Future research should build upon these findings by investigating the influence of organizational characteristics on sustainability measurement and exploring the relationship between the CSAT Short and other sustainability outcomes as well as conducting future cross-setting evaluations.

## Supplementary Information

Additional file 1.

## Data Availability

The deidentified participant data and data dictionary, will be made available in a public repository, specifically Dataverse, after publication of the primary manuscript.

## Abbreviations

CSAT: Clinical Sustainability Assessment Tool
CSAT Short: Clinical Sustainability Assessment Tool 21-item short form
IMPACT: Improving the Management of symPtoms during And following Cancer Treatment
CFA: Confirmatory Factor Analysis
PRO: Patient Reported Outcomes
IRT: Item Response Theory
mIRT: Multidimensional Item Response Theory
CFI: Comparative Fit Index
TLI: Tucker Lewis Index
RMSEA: Root Mean Squared Error of Approximation
SRMR: Standardized Root Mean Residual
WLSMV: Weighted least squares mean and variance

## Footnotes

1 In the final published version of the CSAT Short Form, the phrasing of three items (CSAT7, CSAT15, CSAT20) was revised. The phrasing of these three items as tested in this study are presented in Table 2.
