# Supplementary material for: Psychometric properties of the Clinical Sustainability Assessment Tool (CSAT) short form across three research centers evaluating effectiveness and implementation of a cancer symptom surveillance and management intervention
Source: Implement Sci Commun. 2026 Jan 29;7:41. doi: 10.1186/s43058-026-00867-5 (PMC12934111; doi:10.1186/s43058-026-00867-5)
Supplement: Supplementary file 1 — Additional file 1. [file 43058_2026_867_MOESM1_ESM.docx]

Additional File 1

CFA Reporting Guideline Checklist

| **Information to Report in a CFA Study^1^** | **Page #** |
| --- | --- |
| Model Specification |  |
| • Conceptual/empirical justification for the hypothesized model | 5,8 |
| • Complete description of the parameter specification of the model | 8-12 |
| —List the indicators for each factor | 8 |
| —Indicate how the metric of the factors was defined (e.g., specify which observed variables were used as marker indicators) | 8,12 |
| —Describe all freely estimated, fixed, and constrained parameters (e.g., factor loadings and cross-loadings, random and correlated indicator errors, factor correlations, intercepts and factor means) | Table 5,6; P.12 |
| ––Demonstrate that the model is identified (e.g., positive model df, scaling of latent variables, absence of empirical underidentification) | 12 |
| Input Data |  |
| • Description of sample characteristics, sample size, and sampling method | 7 |
| • Description of the type of data used (e.g., nominal, interval; scale range of indicators) | 8,9 |
| • Tests of estimator assumptions (e.g., multivariate normality of input indicators) | 12 |
| • Extent and nature of missing data, and the method of missing data management  (e.g., direct ML, multiple imputation) | 9 |
| • Provide sample correlation matrix and indicator SDs (and means, if applicable) | Table 3 |
| Model Estimation |  |
| • Indicate the software and version used (e.g., LISREL 8.72) | 9 |
| • Indicate the type of data/matrices analyzed (e.g., variance–covariance, tetrachoric correlations/asymptotic covariances) | 13, table 3 |
| • Indicate the estimator used (e.g., ML, weighted least squares; as justified by properties of the input data) | 9 |
| Model Evaluation |  |
| • Overall goodness-of-fit | 12 |
| —Report model χ2 along with its df and p value | 12 |
| —Report multiple fit indices (e.g., SRMR, RMSEA, CFI) and indicate cutoffs used (e.g., RMSEA ≤ .06); provide confidence intervals, if applicable (e.g., RMSEA) | 12 |
| • Localized areas of ill fit | 12 |
| —Report strategies used to assess for focal strains in the solution (e.g., modification indices/Lagrange multipliers, standardized residuals, Wald tests, EPC values) | 12 |
| —Report absence of areas of ill fit (e.g., largest modification index) or indicate the areas of strain in the model (e.g., modification index, EPC value) | 12 |
| • If model is respecified, provide a compelling substantive rationale for the added or removed parameters and clearly document (improvement in) fit of the modified models | N/A |
| • Parameter estimates |  |
| —Provide all parameter estimates (e.g., factor loadings, error variances, factor variances), including any nonsignificant estimates | Figure 1, Table 2. |
| —Consider the statistical significance of the parameter estimates (e.g., are all indicators meaningfully related to the factors?) | 13 |
| —Ideally, include the standard errors or confidence intervals of the parameter estimates | N/A |
| • If necessary (e.g., suitability of N could be questioned), report steps taken to verify the power and precision of the model estimates (e.g., Monte Carlo evaluation using the model estimates as population values) | N/A |
| Substantive Conclusions |  |
| • Discuss CFA results in regard to their substantive implications, directions for future research, and so on. | 15 |
| • Interpret the findings in context of study limitations (e.g., range and properties of the indicators and sample) and other important considerations (e.g., equivalent CFA models) | 17-18 |

^1^ Brown, T. A. (2015). *Confirmatory factor analysis for applied research*. Guilford publications.
